# Teeth

**Published:** 1857-03

**Authors:** 


### TEETH.

We received,a short time ago,a lot of teeth from the manufactory of Jones, White & McCurdy, which are decidedly the finest teeth we have ever seen. It struck us at first that they had outdone themselves, and we did rather doubt the propriety of producing such rare specimens, so perfect in form, color and life-like tint, for they are calculated to excite admiration and expectation, and then if you don't live up to the point the profession will be disappointed. Well, we will promise that we will not tell how fine they are, nor show them *around* much till we see whether you can do it again.

But to the profession we just have two or three words to say. In these teeth we have the most perfect form, the coloring is faultless and the texture is quite an improvement upon any former specimens.

The peculiar forms for adaptation to plates is excellent; in many cases very little grinding, even of the gum teeth, adapts them perfectly. Another arrangement we find perfected in these, that is beveling the first bicuspid and thickening a little the posterior edge of the cuspids, so that the inside of the circle will be smooth all round. Ordinarily there is a very *objectionable projection* of the first bicuspid past the canine. It is difficult to solder, difficult to finish, and always unpleasant in the mouth, but we will have no more of it now.

Our attention was directed not long since to some French teeth, the texture of which was very fine, and we ventured the assertion that our manufacturers would attain the same excellence, but did not know that it would be done so soon. These teeth WILL stand *fire.*

With these teeth we cannot imagine anything else to be desired, except a large invoice of them at our dental depots.

